# The quality of recovery after erector spinae plane block in patients undergoing breast surgery: a randomized controlled trial

**DOI:** 10.1186/s12871-022-01760-z

**Published:** 2022-07-14

**Authors:** Marcin Wiech, Paweł Piwowarczyk, Marcin Mieszkowski, Bułat Tuyakov, Karolina Pituch-Sala, Tomasz Czarnik, Andrzej Kurylcio, Mirosław Czuczwar, Michał Borys

**Affiliations:** 1 Second Department of Anesthesiology and Intensive Care Medicine, Medical University of Lublin, ul. Staszica 16, 20-081 Lublin, Poland; 2 Department of Anesthesiology and Intensive Care, Regional Specialist Teaching Hospital, Olsztyn, Poland; 3 Department of Anesthesiology and Intensive Care, School of Medicine, Collegium Medicum, University of Warmia and Mazury, Al. Warszawska 30, 10-082 Olsztyn, Poland; 4 Department of Anesthesiology, Intensive Care and Regional ECMO Center, Institute of Medical Sciences, Opole University, Opole, Poland; 5 Department of Surgical Oncology, Medical University of Lublin, ul. Radziwiłłowska 13, 20-080 Lublin, Poland

**Keywords:** Erector spinae plane block, Breast surgery, Quality of recovery, Visual analog scale, Patient-controlled analgesia

## Abstract

**Background:**

The erector spinae plane (ESP) block has recently been shown to effectively alleviate postoperative pain and reduce opioid consumption in breast surgery patients. However, data are still limited concerning the quality of recovery in patients following this procedure.

**Methods:**

This study was a randomized controlled trial (RCT) performed in a university hospital. We randomly allocated patients to one of three groups: ESP, SHAM, and control (CON). Procedures in the ESP and SHAM blocks were performed ipsilaterally with 0.375% ropivacaine or 0.9% saline (0.4 mL/kg). Our primary outcome was the assessment of the patient’s improvement with quality-of-recovery 40 (QoR-40) a day after surgery. Other outcome assessments included postoperative pain evaluation on the visual analog scale (VAS), 24-hour opioid consumption with patient-controlled analgesia (PCA), time to the first opioid demand, and global satisfaction with perioperative treatment.

**Results:**

Overall, patients in the ESP group had improved QoR-40 compared to the CON group, 186 [177–193] vs. 175 [165–183] (medians and interquartile ranges). Pain severity was significantly higher in the CON group compared to the ESP group at hours 2 (38 [23–53] vs. 20 [7–32]) and 4 (30 [18–51] vs. 19 [7–25]). Moreover, we observed lower oxycodone consumption after 24 hours with the PCA pump between the ESP (4 [2–8] mg) and the CON (9.5 [5–19]) groups. Patients in the CON group used PCA sooner than those in the ESP group. Participants in the ESP group were more satisfied with treatment than those in the CON group. We found no statistical difference between SHAM and the other groups.

**Conclusions:**

Compared to the CON group, the ESP block improved the quality of recovery, alleviated pain intensity, and lowered opioid consumption in patients undergoing breast surgery. However, we did not observe this superiority in comparison with the SHAM group.

**Trial registration:**

NCT04726878.

## Background

Regional anesthesia techniques are widely used for breast surgery. For example, thoracic paravertebral block (TPVB) and thoracic epidural anesthesia/analgesia (TEA) have been shown to lower acute postoperative pain, lessen opioid demand, and improve patients’ recovery [1–4].

Recently, researchers have evaluated new regional techniques in patients undergoing breast surgery. In particular, the erector spinae has proven to reduce pain severity and opioid consumption in this group of patients [5]. Further, in a recent meta-analysis, the ESP block was shown to effectively alleviate postoperative pain severity and reduce opioid consumption [6]. However, data are still limited concerning the multidimensional assessment of the quality of recovery in patients following this procedure.

Quality of Recovery 40 (QoR-40) is a multidimensional questionnaire addressing many aspects of postoperative improvement [7]. The QoR-40 has been used numerous times to measure patients’ recovery after different surgeries [8, 9]. In addition, this questionnaire seems to be a reliable tool for assessing other techniques, including regional blocks [10].

Our study aimed to evaluate the quality of recovery in patients undergoing breast surgery. In addition, we measured other outcomes, including postoperative pain severity, opioid consumption, and the time to the first analgesic demand in this population of patients.

## Methods

We conducted the trial after obtaining approval from the Internal Review Board of the Medical University of Lublin (KE-0254/92/2018, chairman Professor M. Olejossy). We registered our study on the ClinicalTrials.gov site on 27/01/2021 with the number NCT04726878 before recruiting patients. Finally, we obtained written informed consent from each patient, and the study was conducted according to the tenets of the Declaration of Helsinki for medical research involving human subjects.

### Patients

For eligibility, we assessed adults (≥ 18 to ≤80 years old) scheduled for single-side breast surgery due to cancer. We excluded patients unable to give informed consent, who had previously participated in the trial (the second breast or a reoperation on the same side), and who qualified for surgery on two breasts. We also disqualified patients with known coagulopathy, allergies to the studied drugs, depression, epilepsy, antidepressant drug treatment, usage of painkillers before surgery, and addiction to alcohol or recreational drugs.

One to 4 weeks before surgery, patients visited our preanesthetic clinic, where an attending anesthesiologist qualified them for anesthesia. An anesthesiologist identified other participants for our study. A meeting was held for the purpose of screening and affirming the patients’ willingness to participate in our trial. A day before surgery, an anesthesiologist who participated in the study discussed with each patient the potential risks and benefits of taking part in the trial. The patients then verified and signed their informed consents to participate in our study. Finally, the anesthesiologist presented and explained the QoR-40 form, the visual analog scale (VAS), and demonstrated the use of the patient-controlled analgesia (PCA) pump. All patients were informed that they could withdraw from the study at any time.

### Anesthesia

We anesthetized the patients participating in the study in a similar manner using fentanyl (Fentanyl, Polpharma S.A., Warszawa, Poland) and propofol (Propofol 1% Fresenius, Fresenius Kabi Deutschland GmbH, Bad Homburg, Germany) to induce general anesthesia. Then, an anesthesiologist inserted a laryngeal mask airway. If the risk of aspiration was high, the anesthesiologist secured the airway with an endotracheal tube. In this case, rocuronium and suxamethonium could be used. We maintained anesthesia with sevoflurane and fentanyl. We emerged patients from general anesthesia using oxygen, sugammadex, or neostigmine, as required. An anesthesiologist assessed the patient before transfer to the postoperative care unit, taking vital signs and applying the Richmond Agitation Sedation Scale.

### Intervention

After inducing general anesthesia, an anesthesiologist involved in the study opened a sealed envelope containing the patient’s allocation. We randomized patients into three groups—the ESP block group (ESP), the sham block group (SHAM), and the control group (CON). We continued general anesthesia in the CON group without modification. Participants were unaware of their allocations.

**Fig. 1:**
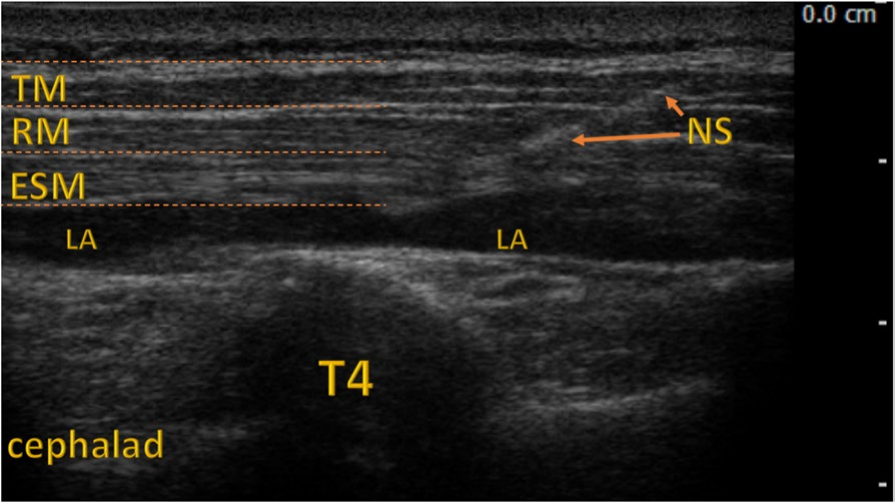
Erector Spinae Plane Block. ESM = erector spinae muscle; LA = site of local anesthetic; NS = needle shaft; RM = rhomboid muscle; T4 = transverse process of the fourth thoracic vertebra; TM = trapezius muscle

Patients in the ESP and SHAM groups were placed in the lateral position contralaterally for injections. The anesthesiologist scanned the patient’s back to determine the injection site (Fig. 1). After preparing the injection field, the ESP block was performed at the level of T4, as shown in Fig. 1. In the ESP group, we used a 0.375% solution of ropivacaine, 0.4 mL/kg, to a maximum of 40 mL administered on the unilateral side. In the SHAM group, we injected 0.4 mL/kg of normal saline into the ESP space up to 40 mL. After injections, we placed the patients supine to perform the surgery.

### Analgesia and postoperative care

Approximately 30 minutes before the end of the surgery, the patient received oxycodone intravenously (IV) at a dose of 0.1 mg/kg. The analgesia regime also included IV Paracetamol, one gram every six hours. In the postoperative care unit, the anesthesiologist initiated the PCA with oxycodone, 1 mg per bolus, with a lockout period of five minutes. If pain exceeded 40 mm on the VAS, the attending nurse could administer a rescue dose of oxycodone (5 mg twice). Routine care included IV Ondansetron, 4 mg twice daily.

### Outcomes

The primary outcome of our study was the result of the QoR-40. We also analyzed parts A and B of the survey. A higher score on the QoR-40 means better recovery following breast surgery. An anesthesiologist who was unaware of participants’ allocations assessed the QoR on the next day following the surgery. We hypothesized that QoR-40 scores in the ESP group would be significantly higher than in the CON group.

Secondary outcomes included postoperative pain severity, opioid consumption, time to the first opioid demand, and treatment satisfaction. An attending nurse not directly involved in the study measured pain severity on the VAS at hours 2, 4, 8, 12, and 24 following surgery. We also assessed overall satisfaction with treatment. Satisfaction was presented on a Likert-type scale from one to five points (*very poor*, *poor*, *moderate*, *good*, and *excellent*). Higher scores indicate greater satisfaction. Moreover, we analyzed the impact of the surgery type—partial resection versus breast amputation—on the aforementioned goals.

### Statistics

We investigated the normality of the distribution for continuous variables with the Shapiro–Wilk test. We analyzed normally distributed parameters using an analysis of variance (ANOVA). These variables are presented as means with 95% confidence intervals. We used the Kruskal–Wallis test by ranks to compute parameters with non-normal distributions. If the Kruskal–Wallis test results showed statistical significance, the Bonferroni correction was applied. Then, a pairwise comparison was performed using the Mann–Whitney U test. These data are presented as medians and interquartile ranges (IQR). Qualitative parameters were compared with Fisher’s exact test. The time to the first demand for oxycodone with PCA was presented as the Kaplan-Meier curve. For this variable, we calculated statistics using the log-rank test. All measurements were performed using Statistica 13.1 software (StatSoft, Tulsa, OK, United States). Randomization was also generated with Statistica software’s random number generator by a team member who was not directly involved in recruiting, treating, and assessing patients.

A preliminary study was performed to assess the sample size. The study’s primary outcome was the quality of recovery measured with the QoR-40. We compared 14 patients, seven after ESP, and seven controls. The mean results of the QoR-40 were 185 after ESP and 172 in patients without any intervention. The calculated sample size was 11 individuals for each group, power 0.8, and alpha 0.05. Because three comparisons were necessary, we decided to randomize 75 participants into groups, with 25 individuals in each group.

## Results

**Fig. 2:**
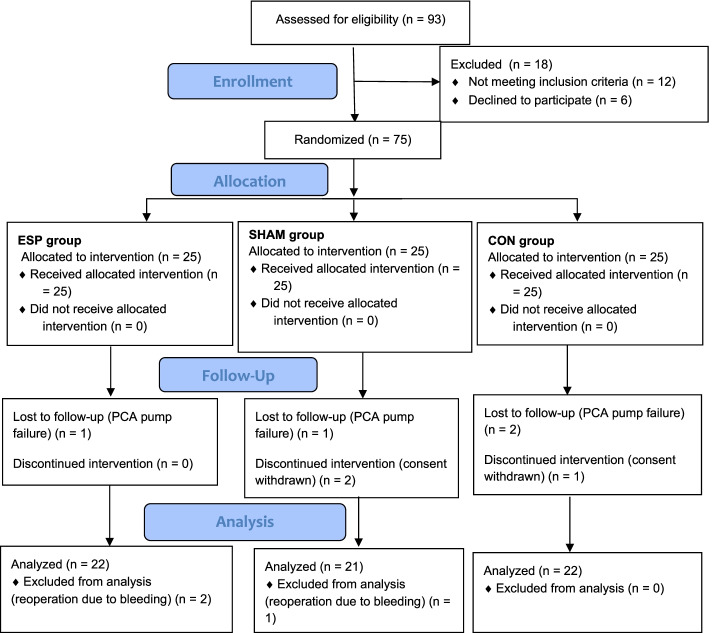
Flowchart. CON = control group, ESP = erector spinae plane group, SHAM = sham block group

The study was conducted from February to August 2021 in the surgical department of the university hospital. We assessed 93 patients for eligibility. Ultimately, we analyzed 65 patients (see Fig. 2, Flowchart). Patient demographics are presented in Table 1. All patients participating in our trial were women.

**Table 1:** Patient demographics and intraoperative period

Groups	CON (***n*** = 22)	SHAM (***n*** = 21)	ESP (***n*** = 22)	***p-***value
Age, years	53.1 (46.8–59.4)	57.1 (50.4–63.8)	56.4 (51.0–61.8)	0.63
Weight, kg	66.8 (61.5–72.1)	70.4 (64.8–76.0)	72.1 (66.2–78.1)	0.35
Height, cm	163 (160–166)	163 (160–166)	165 (161–168)	0.92
BMI, kg/m^2^	25.3 (22.9–27.7)	26.5 (24.4–28.5)	26.7 (24.6–28.9)	0.49
Surgery time, min	132 (113–150)	135 (119–152)	127 (110–144)	0.77
Anesthesia time, min	153 (132–174)	164 (150–178)	162 (144–180)	0.66
LMA/ETT	17/5	19/2	19/3	0.53
Intraoperative Fentanyl, mcg	230 (200–260)	225 (197–253)	211 (192–230)	0.54
Intraoperative fluids, mL	773 (630–915)	667 (543–791)	786 (677–896)	0.32
Surgery side, left/right	12/10	10/11	12/10	0.91
Partial resection/breast amputation	16/6	13/8	16/6	0.75
Sentinel node, yes/no	21/1	21/0	21/1	1.0

Results are presented as means (95% confidence intervals) or n for frequency data. The probability for continuous variables was calculated using one-way ANOVA and frequency data using the Freeman-Halton extension of Fisher’s exact test.

*BMI* body-mass index, *CON* control group, *ESP* erector spinae group, *ETT* endotracheal tube, *LMA* laryngeal mask airway, *N* number of individuals, *SHAM* SHAM group

### Primary outcomes

Patients who received the ESP block scored higher than the CON group on the QoR-40. Moreover, we noticed a difference between the ESP and CON groups for either part A or part B of the QoR-40 (Table 2). The ESP group scored more points than the SHAM group in part B of the QoR-40 questionnaire.

**Table 2:** QoR-40 results

Groups	CON	SHAM	ESP	***p*** value
QoR-40	175 [165–183]^a^	181 [169–188]	186 [177–193]	0.009
QoR-40 A	74 [68–81]^a^	81 [77–83]	83 [75–88]	0.037
QoR-40 B	101 [96–103]^a^	100 [99–104]^b^	104 [103–106]	0.002

Data are shown as medians [interquartile ranges]. Probability was calculated with the Kruskal–Wallis test by ranks. If this test showed a significant result, a pairwise comparison was made with the Mann–Whitney U test. Significant calculated probability was set at 0.017 after the Bonferroni correction.

*CON* control group, *ESP* erector spinae plane group, *SHAM* sham block group, *QoR* quality of recovery.

^a^CON is significantly lower than ESP

^b^SHAM is significantly lower than ESP

### Secondary outcomes

#### Postoperative pain

Acute postoperative pain severity was higher in the CON group than in the ESP group (Table 3) at hours 2 (*p* = 0.012) and 4 (*p* < 0.01).

**Table 3:** Pain severity

Groups	CON	SHAM	ESP	***p*** value
VAS 2	38 [23–53]^a^	28 [19–41]	20 [7–32]	0.031
VAS 4	30 [18–51]^a^	36 [16–46]	19 [7–25]	0.017
VAS 8	25 [20–35]	21 [15–39]	20 [8–22]	0.114
VAS 12	24 [14–43]	21 [14–28]	16 [7–23]	0.071
VAS 24	20 [15–35]	19 [13–29]	17 [6–20]	0.062

Data are shown as medians [interquartile ranges]. Probability was calculated with the Kruskal–Wallis test by ranks. If this test showed a significant result, a pairwise comparison was made with the Mann–Whitney U test. Significant calculated probability was set at 0.017 after the Bonferroni correction.

*CON* control group, *ESP* erector spinae plane group, *SHAM* sham block group, *VAS* visual analog scale.

^a^CON is significantly higher than ESP

#### Opioid consumption

**Fig. 3:**
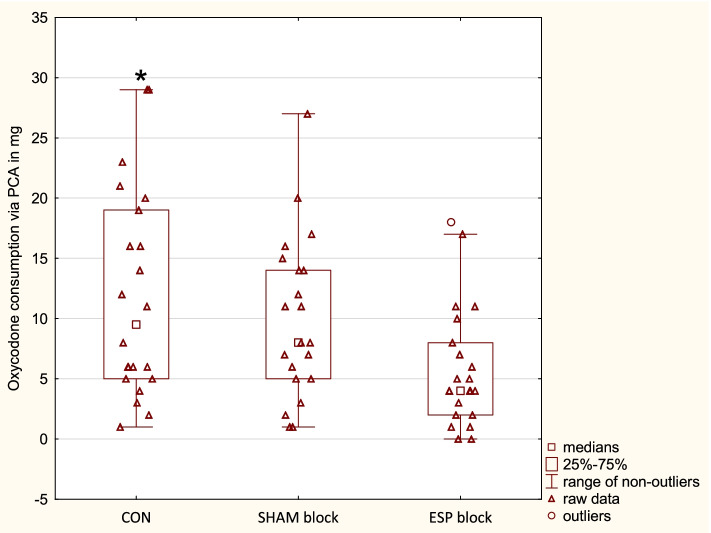
Oxycodone consumption. The figure presents postoperative oxycodone consumption administered with PCA. * CON significantly higher than ESP. CON = control group, ESP = erector spinae plane group, PCA = patient-controlled analgesia, SHAM = sham block group

Postoperative oxycodone consumption (*p* < 0.01) with the PCA pump and all PCA demands (*p* = 0.01) were significantly higher in the CON group than in the ESP group (Fig. 3; Table 4). We found no disparities between the SHAM and the rest of the studied groups. Only five patients required rescue doses of oxycodone—three in the CON group and two in the SHAM group.

**Table 4:** Oxycodone consumption and PCA demands

Groups	CON	SHAM	ESP	***p*** value
Oxycodone consumption via PCA in mg	9.5 [5–19]^a^	8 [5–14]	4 [2–8]	0.014
PCA demands	9.5 [5–23]^a^	11 [6–17]	4 [3–9]	0.021

Data are shown as medians [interquartile ranges]. Probability was calculated with the Kruskal–Wallis test by ranks. If this test showed a significant result, a pairwise comparison was made with the Mann–Whitney U test. Significant calculated probability was set at 0.017 after the Bonferroni correction.

*CON* control group, *ESP* erector spinae plane group, *PCA* patient-controlled analgesia, *SHAM* sham block group.

^a^CON is significantly higher than ESP

#### Treatment satisfaction

Both patients and an assessor found the satisfaction with treatment better in the ESP group than in the CON group (Table 5).

**Table 5:** Treatment satisfaction

Groups	CON	SHAM	ESP	***p***-value
Assessed by patient	4 [4–5]*	5 [4–5]	5 [5–5]	0.001
Assessed by physician	4 [3–4]*	4 [3–5]	5 [5–5]	< 0.001

Data are shown as medians [interquartile ranges]. Probability was calculated with the Kruskal–Wallis test by ranks. If this test showed a significant result, a pairwise comparison was made with the Mann–Whitney U test. Significant calculated probability was set at 0.017 after the Bonferroni correction.

*CON* control group, *ESP* erector spinae plane group, *SHAM* sham block group.

^a^CON is significantly lower than ESP

#### Time to the first PCA demand

**Fig. 4:**
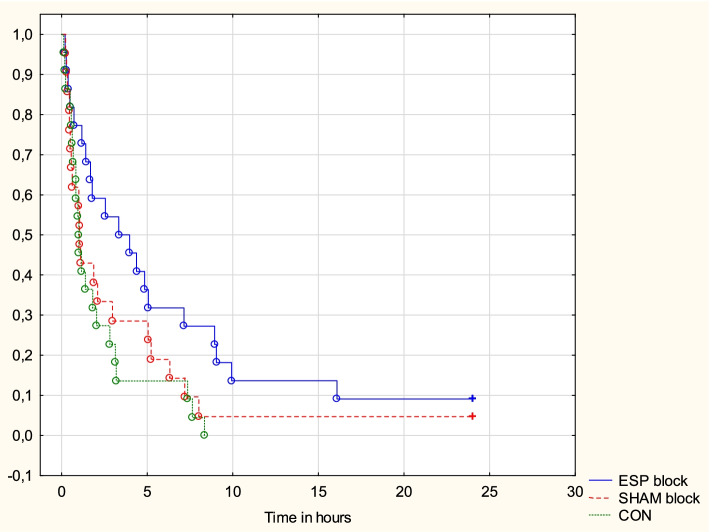
First PCA demand. The figure presents the Kaplan-Meier curve showing first PCA demands. CON = control group, ESP = erector spinae plane group, SHAM = sham block group

Patients in the ESP group used the PCA pump significantly later than participants in the CON group (3.65 [1.18–8.93] vs. 0.98 [0.61–2.81] hour; *p* = 0.014) (Fig. 4). Only three patients, two in the ESP group and one in the SHAM grop, did not use the PCA pump.

#### Surgery type

In all outcomes in the studied patients, we found no disparities between breast amputation and partial resection (Table 6).

**Table 6:** Surgery type

Groups (N)	Partial resection (45)	Breast amputation (20)	***p***-value
QoR-40	180 [168–189]	181 [169–187]	0.61
QoR-40 A	79 [72–84]	80 [72–85]	0.88
QoR-40 B	103 [99–105]	102 [100–103]	0.25
VAS 2	26 [15–42]	30 [17–50]	0.61
VAS 4	21 [16–35]	30 [13–49]	0.31
VAS 8	21 [15–30]	21 [14–38]	0.68
VAS 12	19 [11–27]	19 [11–30]	1.0
VAS 24	18 [11–23]	19 [8–30]	0.65
Oxycodone consumption via PCA in mg	7 [4–14]	6.5 [5.0–15.5]	0.48
PCA demands	7 [4–16]	7.5 [5–24]	0.34

The table presents the study’s outcomes according to surgery type. Data are shown as medians [interquartile ranges]. Probability was calculated with the Mann–Whitney U test.

*PCA* patient-controlled analgesia, *QoR* quality of recovery, *VAS* visual analog scale

## Discussion

The primary hypothesis of our study—patients undergoing breast surgery with the ESP block have better quality of recovery—was confirmed in the results. However, the ESP group achieved better recovery than the CON group, as measured on QoR-40, but not better than the SHAM group. We found a disparity between the ESP group and the CON and SHAM groups only in Part B of the QoR-40 (Table 2).

Patients in the CON group had more severe pain than those in the ESP group, but only in hours 2 and 4 (Table 3). According to this outcome, there was no difference between the ESP and the SHAM groups or the SHAM and the CON groups. As presented in Table 4, patients after the ESP block used less oxycodone and had fewer PCA demands than the CON group. Moreover, the first opioid demand was significantly sooner in the CON group than in the ESP group. Again, we observed no disparity between the SHAM and the other groups. Finally, the surgical technique, total versus partial breast resection, did not impact outcomes in our study (Table 6).

To our knowledge, the only study in which the ESP block was compared with a SHAM block was by Yao et al. [11]. Moreover, Yao et al. measured the quality of recovery of patients following breast surgery. However, the authors of this trial used a shorter version of QoR-40, QoR-15. In contrast with our results, they reported a significant difference between the two groups in favor of the ESP group, according to each of the primary outcomes, including quality of recovery, pain severity, and opioid consumption. We observed a disparity between the ESP and the CON groups in the current study, but not between the SHAM and the other groups. It is possible that the lack of statistical difference between the ESP and the SHAM groups was caused by the inclusion of a third group in our trial.

Our results concerning postoperative pain severity are consistent with most trials comparing the ESP block with a CON group. The ESP alleviated pain intensity after breast surgery measured with VAS or a numerical rating scale [5, 12–16]. However, Aksu et al. did not observe a difference in pain severity between the ESP block and the CON, but morphine consumption was lower after the regional block [17]. In this study, the ESP block was performed at two levels; thus, the total volume of injection was divided. This maneuver could change the spread of local anesthetic. As presented in the cadaveric study by Choi et al., a larger injection volume during the ESP block results in a vaster area covered with dye [18].

The results of each study comparing the ESP block to a CON group showed that this regional block reduced opioid consumption in the postoperative period [5, 12–17]. However, the PECS block could be even more effective in reducing pain severity and opioid consumption after breast surgery than the ESP block, as presented in studies by Sinha et al. and Altiparmark et al. [19, 20].

We did not find significant differences between the ESP and the SHAM groups in the current study. As mentioned above, our study may be underpowered to present disparities between these groups, and more participants would be required. However, it is possible that some pain-relieving action of the ESP block is caused by spreading the fluid and separating fasciae. Especially the long-term pain relief in patients following the ESP or other plane blocks cannot be explained by local anesthetic action only [21, 22].

Our study has some limitations. First, although we calculated the sample size according to the preliminary results, the studied population was still small. Second, the group was heterogeneous due to two types of surgeries. Third, we did not check the ESP block area with the pinprick technique because the patient was anesthetized generally.

## Conclusions

To conclude, the results presented in our study showed the superiority of the ESP block over a CON group in the quality of recovery in patients undergoing breast surgery. Moreover, the ESP block lessened pain severity and reduced opioid consumption. However, we did not prove the advantage of the ESP block over the SHAM group in any studied outcomes.

## Data Availability

The data are available from the corresponding author after a reasonable request.

## Abbreviations

CON: Control Group
ESP: Erector Spinae Plane
IV: Intravenously
IQR: Interquartile Ranges
TPVB: Paravertebral Block
PCA: Patient-Controlled Analgesia
PECS: Pectoral Nerves
QoR-40: Quality of Recovery 40
TEA: Thoracic Epidural Anesthesia/analgesia
VAS: Visual Analog Scale
